# Early detection of sepsis using artificial intelligence: a scoping review protocol

**DOI:** 10.1186/s13643-020-01561-w

**Published:** 2021-01-16

**Authors:** Ivana Pepic, Robert Feldt, Lars Ljungström, Richard Torkar, Daniel Dalevi, Hanna Maurin Söderholm, Lars-Magnus Andersson, Marina Axelson-Fisk, Katarina Bohm, Bengt Arne Sjöqvist, Stefan Candefjord

**Affiliations:** 1 Department of Electrical Engineering, Chalmers University of Technology, Gothenburg, 412 96 Sweden; 2 Department of Computer Science and Engineering, Chalmers University of Technology, Gothenburg, 412 96 Sweden; 3 Department of Infectious Diseases, Institute of Biomedicine, Sahlgrenska Academy, Gothenburg University, Gothenburg, Sweden; 4 Region Västra Götaland, Skaraborg Hospital, Department of Infectious Diseases, Skövde, Sweden; 5 Aweria AB, Gothenburg, 411 18 Sweden; 6 PreHospen Centre for Prehospital Research, University of Borås, Borås, 50 190 Sweden; 7 Department of Mathematical Sciences, Chalmers University of Technology, Gothenburg, 412 96 Sweden; 8 Karolinska Institute, Department of Clinical Science and Education, South General Hospital, Stockholm, Sweden; 9 Department of Emergency medicine, South General Hospital, Stockholm, Sweden; 10 MedTech West, Sahlgrenska University Hospital, Gothenburg, 413 45 Sweden

**Keywords:** Sepsis, Artificial intelligence, Machine learning, Prehospital care, Emergency department, Clinical decision support

## Abstract

**Background:**

Sepsis is a life-threatening organ dysfunction caused by a dysregulated host response to infection. To decrease the high case fatality rates and morbidity for sepsis and septic shock, there is a need to increase the accuracy of early detection of suspected sepsis in prehospital and emergency department settings. This may be achieved by developing risk prediction decision support systems based on artificial intelligence.

**Methods:**

The overall aim of this scoping review is to summarize the literature on existing methods for early detection of sepsis using artificial intelligence. The review will be performed using the framework formulated by Arksey and O’Malley and further developed by Levac and colleagues. To identify primary studies and reviews that are suitable to answer our research questions, a comprehensive literature collection will be compiled by searching several sources. Constrictions regarding time and language will have to be implemented. Therefore, only studies published between 1 January 1990 and 31 December 2020 will be taken into consideration, and foreign language publications will not be considered, i.e., only papers with full text in English will be included. Databases/web search engines that will be used are PubMed, Web of Science Platform, Scopus, IEEE Xplore, Google Scholar, Cochrane Library, and ACM Digital Library. Furthermore, clinical studies that have completed patient recruitment and reported results found in the database ClinicalTrials.gov will be considered. The term artificial intelligence is viewed broadly, and a wide range of machine learning and mathematical models suitable as base for decision support will be evaluated. Two members of the team will test the framework on a sample of included studies to ensure that the coding framework is suitable and can be consistently applied. Analysis of collected data will provide a descriptive summary and thematic analysis. The reported results will convey knowledge about the state of current research and innovation for using artificial intelligence to detect sepsis in early phases of the medical care chain.

**Ethics and dissemination:**

The methodology used here is based on the use of publicly available information and does not need ethical approval. It aims at aiding further research towards digital solutions for disease detection and health innovation. Results will be extracted into a review report for submission to a peer-reviewed scientific journal. Results will be shared with relevant local and national authorities and disseminated in additional appropriate formats such as conferences, lectures, and press releases.

**Supplementary Information:**

The online version contains supplementary material available at (10.1186/s13643-020-01561-w).

## Background

Since 2016, sepsis is defined as “life-threatening organ dysfunction caused by a dysregulated host response to infection” [1]. This definition replaces the previous definition from 1992 based on markers of systemic inflammation [2]. The new definition of sepsis also replaces the old term “severe sepsis,” which was used to designate organ dysfunction caused by infection. The new definition provides a more accurate understanding of the pathophysiology of sepsis as well as more precise diagnostic criteria. Sepsis accompanied by circulatory failure is termed septic shock, the most severe form of sepsis [3]. Together with the definition of sepsis, the recommendation for making a definitive diagnosis changed to “appropriate routine microbiologic cultures (including blood) be obtained before starting antimicrobial therapy in patients with suspected sepsis or septic shock if doing so results in no substantial delay in the start of antimicrobials” [4].

Using the old definition of sepsis and severe sepsis, an assessment of global incidence and mortality of hospital-treated sepsis including years 2003–2015 found that sepsis and severe sepsis constituted 17% and 26% of all in-hospital deaths, respectively, summing up to 5.3 million deaths annually [5, 6]. In 2008, 2% of the overall number of hospitalizations in the USA were caused by sepsis, which led to 17% of in-hospital deaths [5]. Every year the number of deaths from sepsis in the USA amounts to 250,000 and affects around 1.5 million people [7]. Total nationwide US cost of treating these patients was estimated to $14.6 billion in 2008 [5]. Using the new definition, the annual incidence of sepsis in Sweden was estimated to 838/100,000, which is 3-fold higher than that of severe sepsis [8]. The case fatality rate for sepsis is at least 10% and for septic shock at least 40% [3, 9]. This is considerably higher than e.g. for acute myocardial infarction, which is around 5%. The sepsis case fatality rate is highly age-dependent and increases with higher age [10, 11].

Sepsis is a leading cause of mortality in hospitals [12]. Each hour of delay is associated with reduction in patient survival, but studies show that delays are not uncommon in hospitals [13–15]. A number of early warning systems have been developed in order to improve survival outcomes, such as National Early Warning Score (NEWS) and Quick Sepsis-Related Organ Failure Assessment (qSOFA) [16, 17]. NEWS showed good performance in identifying patients at risk of cardiac arrest, deterioration, unexpected intensive care unit (ICU) admission, or death, but also the need for hospital admission [16, 18]. Scoring systems have a role as a tool to predict the need for surgical admission and likely outcomes, especially in severe illness where the signs could be missed, like sepsis [16]. The early recognition of deteriorating physiological parameters can possibly provide earlier, more effective intervention [12]. Timely administration of antibiotics and treatment of sepsis patients can make a significant difference in outcome, and early warning scores may help form a pre-alert protocol or indicate specific prehospital treatments [16].

Early detection and prompt intervention play a key role in optimizing the outcome of sepsis patients [19]. More timely identification and management is required for patient outcomes to improve. Positive outcomes are highly related to effective management in prehospital settings and emergency departments (ED), since successful treatment is time-dependent [20]. Although recommendations for management of the patients are suggesting consideration of “golden hour” and “silver day,” representing the first few hours of disease presentation and the few remaining hours of the first day, respectively, transferring the patient from the ED to an ICU is often not performed in a timely manner [21–23]. Clinicians face a challenge in differentiating sepsis from other acute conditions due to similarities with signs or symptoms for other common diseases.

Artificial intelligence (AI) has the potential to deliver timely and accurate sepsis detection [24, 25], potentially outperforming current clinical warning scores, which are not based on sophisticated mathematical models. Early prediction of sepsis could be achieved by developing a decision support system based on machine learning (ML) algorithms trained on patient data, usually based on electronic medical records, biomedical signals, and/or laboratory results [26–28]. In this scoping review, we will analyze the literature on AI methods for early detection of sepsis.

The terms AI and ML are difficult to define clearly, and there is considerable variation in how different authors use the terms. While, for example, McCarthy [29], Domingos [30], and Bini [31] view AI very generally while considering ML as a subset of it, other texts [32] take a more statistical perspective and simply discuss learning from data without making an explicit distinction between terms. We do not take a particular stance on this. Rather, our goal is to be inclusive and to view any kind of (mathematical) model that is suitable for digitization and has potential to improve detection accuracy of sepsis, as relevant for our review. In this study, when we refer to AI or ML, we refer to a broad range of mathematical models.

Similar to the reasoning about what AI and ML encompass, the terminology for referring to methods able to predict that a patient is at high risk of having sepsis varies. Terms like “detect”, “identify,” “predict,” “recognize,” and their variations (detection, etc.), along with other words such as “infer,” appear to be used interchangeably in the literature. We will distinguish terms like “detection” and counterparts from “diagnosis,” where the latter in our experience should denote a clinical standard for establishing that a patient suffers from sepsis (which should include microbiologic cultures as per above recommendation). We will use “detect” and its synonyms for referring to predicting high risk of sepsis.

To manage that studies will use different definitions of sepsis, e.g., studies prior to the introduction of the Sepsis-3 definition in year 2016 will not use the current standard, studies will be grouped into different categories as deemed suitable according to the numbers of studies that will be included in each group.

### Study aim

The overall aim of this scoping review is to summarize the literature on existing methods for early detection of sepsis using AI. We define early detection as occurring during the prehospital assessment or in the ED. We have established the following objectives to fulfil the overall aim:

1. Provide a summary of state-of-the-art approaches to use AI (viewed broadly) to detect sepsis during the prehospital phase and/or in the ED. The summary will focus on diagnostic accuracy and perceived clinical usability and discuss ethical robustness of the methods.
2. Recognize the most commonly used clinical protocols for patient screening and early warning of possible sepsis. Their diagnostic accuracy and usability will be compared to the findings for emerging methods based on AI.
3. Identify predictor variables stemming from patient data and other data sources commonly used in AI methods for early sepsis detection. Discuss whether any predictor variables appear to be especially important.
4. Recognize challenges, weaknesses, and establish unaddressed issues that can help improve future research and innovation in this area.

## Method

A scoping review is identified by Arksey and O’Malley [33] as a type of literature review that aims to “map” relevant literature in the research field being addressed. It differs from systematic reviews and meta-analyses in that typically broader topics will be covered, which allows for a wider range of study designs to be included [33]. The present study will be performed by following the model by Arksey and O’Malley, with further clarifications and recommendations to the framework by Levac et al. [34]. The model defines a six-stage methodological framework, which includes identifying the research question, identifying relevant studies, study selection, charting the data, and collating, summarizing, and reporting the result. A consultation exercise is an optional last stage that will not be included in the present study due to the experimental nature of the reviewed methods, making it difficult for practitioners to provide judgment, and due to time restrictions to complete the review. Three of the authors are clinical experts on infection/sepsis and emergency care; they will assist in providing insight into the potential for clinical usability. The scoping review protocol is being reported in accordance with the reporting guideline provided in the Preferred Reporting Items for Systematic Reviews and Meta-analyses protocols (PRISMA-P) statement. PRISMA-P checklist is developed for the systematic review protocol and therefore not all items will be covered (see Additional file 1: PRISMA-P checklist).

### Stage 1: Identifying the research question

A preliminary review of the literature on early detection of sepsis using AI/ML and related mathematical models was carried out in order to better refine the scope of this protocol. The following research questions to be addressed were identified.

1. Which steps are in general present and necessary for developing AI, ML, or statistical methods for detecting sepsis in prehospital settings or in the ED?
2. What are the predictor variables that are most often used and appear to be necessary for accurate early identification of sepsis using AI?
3. What is the accuracy of the included algorithms for sepsis detection? It should be quantified by suitable measures such as sensitivity, specificity, and the area under the receiver operating characteristic curve. It should be evaluated by some form of out-of-sample accuracy estimation, such as *n*-fold cross-validation or using a validation set never used for training the classifier.
4. Do any prospective studies that evaluate the performance of an AI method on a new, unseen cohort of sepsis patients exist? If yes, how do the reported accuracies compare to retrospective studies using AI, and to traditional early warning systems in clinical use?
5. What are the current challenges and limitations of the reviewed methods, and what are the possible issues that needs to be addressed to develop a tool suitable for clinical use?

### Stage 2: Identifying relevant studies

The goal of the second stage of the scoping review is to identify primary studies and reviews that are suitable to answer our research questions. To be able to accomplish that, we will follow an elaborate strategy for scoping a broad spectrum of literature following defined criteria, and implementing filters that will help us refine the search for relevant studies. When the initial literature search has been completed, we will use a so-called snowball approach to find additional relevant studies, following the guidelines by Wohlin [35].

A comprehensive literature collection will be compiled by searching several sources, including electronic databases and reference lists. Some constricts have to be implemented due to time and language limitations. Therefore, only studies published between 1 January 1990 and 31 December 2020 will be taken into consideration. To our knowledge, there are no relevant studies before the period included here, due to changes over time of study protocols, sepsis definitions, and AI/ML technology. Foreign language publications will not be considered due to time and cost of translating documents, i.e., only papers with full text in English will be included. We are aware that this means that relevant papers could be missed. Search terms are developed to make a full coverage on the topic, including a broad notion of different methods.

#### Information sources

##### Electronic databases, the Internet, and research registers

For this study, the following electronic databases/web search engines will be used: PubMed, Web of Science Platform, Scopus, IEEE Xplore, Google Scholar, the Cochrane Library, and the ACM Digital Library (for a more detailed list, please see Additional file 2: Databases Covered and used Search Engines). Eligible clinical studies that have completed patient recruitment and reported results found on ClinicalTrials.gov will be considered as well. Hand-searching of key journals will not be performed as we deem that there is enough coverage from electronic databases, combined with the snowballing approach, to provide a comprehensive overview of the researched topic.

Selecting appropriate key search terms is essential. The keywords that will be used include sepsis, septic shock, diagnosis, detect, identify, predict, infer, prehospital, emergency department, artificial intelligence, neural networks, deep learning, machine learning, decision support, statistic, mathematical, model. Further search terms may be added, as deemed necessary to cover the intended scope comprehensively [35]. Retrieved articles will be screened for their titles, abstracts, and index terms in mentioned databases. After defining search strings for each database/search engine (using the same search terms, adapting the string as suitable for each service), articles will be retrieved from the database and imported into an open-source reference management software called Zotero [36]. Search strategy developed for PubMed database is presented in Table 1 as an example. We will use Zotero’s built-in functionality for eliminating duplicate items retrieved from multiple services.

**Table 1:** Search strategy developed for PubMed database

PubMed		
Date of search	25 September 2020	
Time limitation	1 January 1990 and 30 September 2020	
Search string	1	sepsis
		septic shock
	2	diagnosis
		detect
		identify
		predict
	3	prehospital
		emergency department
	4	artificial intelligence
		neural networks
		deep learning
		machine learning
		decision support
		statistic
		mathematical model
Number of results	441	

Search words are separated into groups according to key concepts for the scoping where relationship between them (search terms on consecutive lines) corresponds to boolean OR operator. For every concept, at least one search term needs to be present in every article.The complete search string will be constructed from substrings 1 to 4 using the boolean AND operator

At the beginning of the scoping process, and also during the review process, the team will meet to discuss decisions, challenges, or uncertainties related to study inclusion and exclusion. The search strategy will be refined as needed, depending on the abstracts obtained from the search.

##### Snowballing, exhaustive searching of reference and citation lists

From the start set of papers found by searching the selected services and applying eligibility criteria to decide final inclusion of each paper, we will use the snowballing approach described by Wohlin [35] to identify further publications that can become new candidates for inclusion. This approach employs backward and forward snowballing, where the reference list of each included article and studies citing the article are explored, respectively. The exploration is performed by successively assessing the title, abstract, and full text of papers and, in each step, deciding whether to reject the paper or explore it further. The final inclusion of a paper is based on a review of the full text and applying eligibility criteria. After a new paper has been included, the forward and backward snowballing procedures is repeated on that paper, and this process continues iteratively until no new publications are found. For full details, please refer to Wohlin [35].

Considering the type of publication, peer-reviewed journal articles and peer-reviewed conference papers will be included in eligibility criteria. Papers addressing detection of sepsis in the prehospital and/or in the ED phases of patient management will be considered. Based on the initial exploratory research and first stage of the scoping review, articles focusing on detection of sepsis in the ICU or after patients have moved out of the ED (discharged home, expired or admitted into the hospital as inpatients) will be excluded. Conference abstracts, book reviews, commentaries, and editorial articles will be excluded.

### Stage 3: Study selection

There is a need for a systematic method to provide consistency in decision-making regarding which articles to include in the study after the initial searches in the second stage have been completed, and analogously for deciding what papers to include from the snowballing approach. To eliminate a large number of irrelevant studies, it is helpful to develop inclusion and exclusion criteria. Included studies need to answer relevant research questions defined in the first stage of this protocol. During the initial screening stage, a large set of articles will be retrieved. For inclusion and exclusion criteria to be developed, familiarity with the literature is needed, and for that reason, an exploratory review was performed initially. Eligibility criteria for this study needs to address information related to type of study, type of method, type of evaluation for classification accuracy, and relevant patient group. In case the article is not written in English language, it will not be considered. Additionally, if the study does not provide a solution intended for early detection of sepsis using AI/ML and is not fully automatic or there is no quantitative validation using a suitable method and metrics, the paper will be excluded. Search for articles to be included in the study analysis will be stopped when all publications from the initial search in stage 2, as well as publications found from snowballing, are exhausted and each candidate has been assessed by the inclusion and exclusion criteria. For inclusion and exclusion criteria list, please see Additional file 3: Eligibility criteria. Final inclusion will be determined based on the analysis of the full text of each paper.

Regarding selecting studies with relevant patient groups, we will use both the new definition for sepsis [1] and the older definitions. Most of the patients with severe sepsis according to the old definition will also have Sepsis-3 [37]. However, the incidence of Sepsis-3 is higher than that of severe sepsis [8]. If appropriate and a sufficient number of studies are included, studies may be grouped into different types according to the sepsis definition used. The impact of the old versus the new sepsis definition will also be discussed.

Following the recommendations by Levac et al. [34], at least two researchers will independently review the full articles to decide if the article should be included in the review or not. The decisions from the researchers will be concealed until all intended reviews have been completed, to avoid that reviewers are biased from awareness of other reviewers’ decisions. If there is disagreement between the researchers about whether to include a study, an additional reviewer will be consulted to decide final inclusion. Krippendorff’s *α*_*k*_ will be calculated to estimate the initial inter-rater agreement. Compared to other statistics, *α*_*k*_ has the following characteristics:

- Evaluates the agreement between ≥2 independent reviewers performing independent analyses
- Utilizes the distribution of the categories or scale points that are used by the reviewers
- Applies a numerical scale between two points, enabling a sensible reliability interpretation
- Is pertinent to the level of measurement of the data
- Has known, or computable, sampling properties

For a more in-depth example of applying *α*_*k*_, please see [38]. When the inter-rater agreement is deemed acceptable, the study selection procedure will be completed.

### Stage 4: Charting the data

This stage will be conducted in order to extract key information and categorize and sort the data accordingly. Study information that will be included in data extraction of every recorded article is:

1. Author(s), year of publication, study location, if available funding information
2. Aims/purpose of the study
3. Study populations, patient database(s), number/types of class labels used for classification
4. Methodology/methods, where we expect to find information in the following groups:
  - Clinical diagnosis of the patient, time to diagnosis, place of diagnosis, time to start of the treatment
  - Data processing methods and procedures
  - Detection methods, computational and mathematical theories applied
  - Validation method
5. Main findings and outcome measures, such as accuracy measured as the area under the receiver operating characteristic curve
6. Limitations of the study stated by its authors

At least two members of the team will test the framework on a sample of included studies to ensure that the coding framework is suitable and can be consistently applied. If needed, the charting categories will be modified and the data extraction framework revised accordingly. The responsible team members will independently chart the data from each included study following the data extraction framework. In order to ensure inter-rater reliability, a sample of the included articles that are in this way independently reviewed will then be compared. A discussion between the reviewers will follow until consensus is reached, or by arbitration of one or more additional reviewers if required.

### Stage 5: Collating, summarizing, and reporting the results

In this stage, we will summarize the results and present an overview of the reviewed literature. Findings will be presented in an adequate way to capture the previously defined list in stage 4 for all included studies, together with the description needed to organize key findings thematically. Analysis of collected data will provide a descriptive summary and thematic analysis. It will contain common characteristics of included studies, collected following a consistent approach for every study. Following such approach, we will be able to make comparisons between included studies, especially used methods, and identify research gaps. Data necessary to detect sepsis using AI will be recognized and key findings presented. Additional details will be included in order to assist in understanding the studies and performing a complete analysis.

The reported results will provide information about the state of current research for using AI to provide early detection of sepsis. Low level evidence indicative on the possibility to detect sepsis during the prehospital phase or while a patient is in the ED can be gathered. The results should yield recognition of where the research gaps are in existing literature. Therefore, summarizing and disseminating research findings will be provided to policy makers, practitioners, and consumers who might otherwise lack time or resources to undertake such research.

## Ethics and dissemination

Ethical approval is not needed as the study will contain information gathered from already published papers. The review should aid in further research towards digital solutions for disease detection and health innovation. Findings and results will be extracted into a review report for submission to a peer-reviewed scientific journal. Results will be published and shared with relevant networks, local and national organizations operating in the field of digital health. Results will be disseminated in appropriate formats such as journal articles, lectures, conferences, and press releases. Amendments to this protocol, if any, will be listed in the final review publication.

## Supplementary Information

**Additional file 1** PRISMA-P+checklist.

**Additional file 2** Databases Covered and used Search Engines.

**Additional file 3** Eligibility criteria.

## Data Availability

Data sharing is not applicable to this article as no datasets were generated or analyzed during the current study.

## Abbreviations

AI: Artificial Intelligence
ED: Emergency Department
ICU: Intensive Care Unit
ML: Machine Learning
NEWS: National Early Warning Score
qSOFA: Quick Sepsis-Related Organ Failure Assessment
