# Supplementary material for: Early detection of sepsis using artificial intelligence: a scoping review protocol
Source: Syst Rev. 2021 Jan 16;10:28. doi: 10.1186/s13643-020-01561-w (PMC7811741; doi:10.1186/s13643-020-01561-w)
Supplement: Supplementary file 2 — Additional file 2 Databases Covered and used Search Engines. [file 13643_2020_1561_MOESM2_ESM.pdf]

Databases Covered and used Search Engines

September 2020

1 Web of science Platform

1.1 Citation Indexes

Web of Science Core Collection (Citation Indexes:

· Science Citation Index
· Social Sciences Citation Index
· Arts Humanities Citation Index
· Conference Proceedings Citation Index
· Book Citation Index
· Emerging Sources Citation Index)

BIOSIS Citation Index
Chinese Science Citation Database
Data Citation Index
Russian Science Citation Index
SciELO Citation Index
Arabic Citation Index

1.2 Subject specialized and regional indexes:

Biological Abstracts, BIOSIS Previews
CABI: CAB Abstracts and Global Health
FSTA—the food science resource
Inspec
KCI—Korean Journal Database
Medline
Zoological Record

1.3 Other resources:

Current Contents Connect
Derwent Innovations Index (Patents)

2 Cochrane Library

Cochrane Database of Systematic Reviews
Cochrane Central Register of Controlled Trials (CENTRAL)
Cochrane Clinical Answers
Featured content:

- · Special Collections
- · Epistemonikos

3 PubMed

PubMed facilitates searching across several U.S. National Library of Medicine (NLM) literature resources:
MEDLINE
PubMed Central (PMC)
Bookshelf

4 IEEE Xplore

5 ACM Digital Library

6 ClinicalTrials.gov

7 Scopus

8 Google Scholar
