# Supplementary material for: Early detection of sepsis using artificial intelligence: a scoping review protocol
Source: Syst Rev. 2021 Jan 16;10:28. doi: 10.1186/s13643-020-01561-w (PMC7811741; doi:10.1186/s13643-020-01561-w)
Supplement: Supplementary file 3 — Additional file 3 Eligibility criteria. [file 13643_2020_1561_MOESM3_ESM.pdf]

Eligibility criteria

September 2020

Inclusion criteria

The following inclusion criteria need to be fulfilled by all of the studies eligible for selection:

- Study specifies information about the type of study (e.g. a randomized controlled trial), type of method, and type of evaluation for classification accuracy.
- Study reports an estimate of the classification accuracy, e.g. using the area under the receiver operating characteristic (ROC) curve (AUC), sensitivity and specificity, or other appropriate measures that indicate potential clinical usefulness
- A relevant patient group is assessed. Both studies using the Sepsis-3 definition and older definitions of sepsis will be included.
- Published peer-reviewed journal articles and peer-reviewed conference papers.
- Written in English language.
- Written during the time period defined in the protocol.

Exclusion criteria

Studies that meet the following exclusion criteria will be excluded:

- Full-text articles cannot be obtained.
- The method presented in the study is not intended for early detection of sepsis.
- The method is not using Artificial Intelligence/Machine Learning.
- The method is not fully automatic.

- There is no quantitative validation of the solution using a suitable method and metrics providing estimates with low bias, e.g. by not performing a cross-validation or by other means estimating prospective performance of the algorithm.
- Conference abstracts, book reviews, commentaries, and editorial articles.
